# Shear-band affected zone revealed by magnetic domains in a ferromagnetic metallic glass

**DOI:** 10.1038/s41467-018-06919-2

**Published:** 2018-10-24

**Authors:** L. Q. Shen, P. Luo, Y. C. Hu, H. Y. Bai, Y. H. Sun, B. A. Sun, Y. H. Liu, W. H. Wang

**Affiliations:** 1 Institute of Physics, Chinese Academy of Sciences, 100190 Beijing, China; 2 University of Chinese Academy of Sciences, 100049 Beijing, China; 3 Beijing Advanced Innovation Center for Materials Genome Engineering, 100083 Beijing, China

## Abstract

Plastic deformation of metallic glasses (MGs) has long been considered to be confined to nanoscale shear bands, but recently an affected zone around the shear band was found. Yet, due to technical limitations, the shear-band affected zone (SBAZ), which is critical for understanding shear banding and design of ductile MGs, has yet to be precisely identified. Here, by using magnetic domains as a probe with sufficiently high sensitivity and spatial resolution, we unveil the structure of SBAZs in detail. We demonstrate that shear banding is accompanied by a micrometer-scale SBAZ with a gradient in the strain field, and multiple shear bands interact through the superimposition of SBAZs. There also exists an ultra-long-range gradual elastic stress field extending hundreds of micrometers away from the shear band. Our findings provide a comprehensive picture on shear banding and are important for elucidating the micro-mechanisms of plastic deformation in glasses.

## Abstract (web summary)

Metallic glasses deform along nanoscale shear bands, and while it is known that they affect the neighboring glass regions, exactly how is unclear. Here, the authors use magnetic force microscopy to atomically resolve the shear-band affected zone and show its effects extends much further than previously thought.

## Introduction

Although metallic glasses (MGs) exhibit remarkable strength and elasticity, ductility has long been the Achilles’ heel that hinders their broad applications as structural materials. Due to strain softening, plastic deformation of MGs at temperatures well below glass transition is strongly localized into shear bands which governs the yielding and fracture behavior of MGs. A shear band is often considered as a nanoscale planar object resulting from cooperative alignment of shear transformation zones (STZs)^1,2^. Previous observations by transmission electron microscopy (TEM) suggest that the thickness of a shear band is approximately 10–20 nm, and this value has long been adopted in many models for MG deformation^1,3^. However, later studies through different techniques imply that a much wider region is involved upon shear banding. For example, an estimate indicates that the stored energy in a deformed MG is too large to be attributed to a 20 nm-thick shear band^4^. The enhanced diffusion and excess contribution to the boson heat capacity peak in a deformed MG have been suggested to be associated with materials surrounding shear bands^5,6^. The width of the heat affected zone around a shear band is much larger^1,7^, and the liquid-like layer in the vicinity of a shear band can be as thick as a few micrometers^3,8^. In addition, radiotracer diffusion^5,9^, nanoindentation^10–13^, X-ray photon correlation spectroscopy^14^, and nanobeam X-ray diffraction^15,16^ all imply a wider region is affected rather than the nanoscale shear band itself. Nonetheless, a variety of values on the width of the affected zone around a shear band have been deduced, ranging from submicrometer to hundreds of micrometers by different techniques^10–17^. A consensus on the effective thickness of shear bands is yet to be reached^1^. These inconsistencies impede a comprehensive understanding of shear banding and plastic deformation of MGs. To precisely map the shear-band affected zone (SBAZ), an approach that is of sufficiently high sensitivity and spatial resolution is required.

It is known that magnetic domains are associated with magnetic anisotropies and reflect the configuration of spins. They are formed to minimize the total magnetic energy of ferromagnet^18^. Originating from spin-orbit coupling, magnetic moments of the domains is coupled to atomic displacements through magneto-elastic coupling or the so-called inverse magnetostriction effect^19,20^. Local rearrangement of atoms can induce reorientation of magnetic moments to minimize the magnetic energy, leading to redistribution of magnetic domains. Based on magneto-elastic coupling, it has been demonstrated that magnetic domain walls can be regulated by straining^21–23^, and atomic displacements due to magnetostriction can be measured even on femtometer-scale^20,24,25^. Deformation strains on the order of ~10^-5^ can hardly be measured by X-rays, but can completely reorder a domain pattern^26^.

For ferromagnetic MGs that are free of magnetocrystalline anisotropy, magnetic domain structures are dominated by magnetoelastic anisotropy and are extremely sensitive to atomic displacements induced by local stress^19,26^. Therefore, the evolution of magnetic domain configurations can directly reflect local structural changes and stress/strain distribution upon deformation of ferromagnetic MGs. That is, magnetic domains that are readily observable by magnetic force microscopy (MFM) can be used to explore, with high precision and high spatial resolution down to nanoscale^27,28^, the affected zones around shear bands.

In this article, we report the measurement of magnetic domains around shear bands upon deformation of Fe-based MGs. The SBAZs for various shear bands, corresponding to different magnetic domain structures are accurately unveiled. We found the formation of wave-like magnetic domains in the vicinity of a single shear band, zipper-like domains extending far from the shear-band core, and paired magnetic domains in-between multiple shear bands. The magnetic domain configurations indicate a multilayer-like structure of the SBAZ with different length scale, and shear bands interact due to the overlap of SBAZs. By directly visualizing SBAZs, our results explain the reasons for the various reported widths of SBAZs in previous investigations and are important for understanding the nature of shear banding, shear band propagation and interaction governing deformation and ductility of MGs.

## Results

### Affected zone around a single shear band

**Fig. 1:**
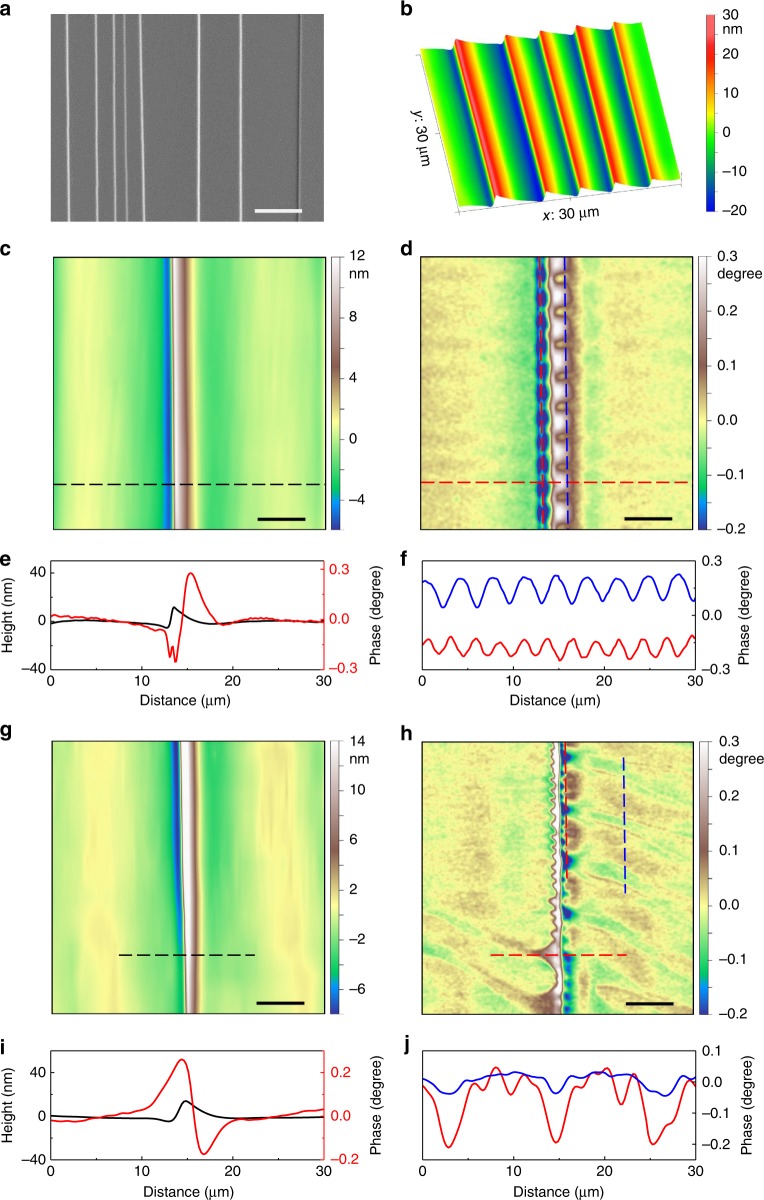
Typical AFM and MFM micrographs of shear bands. **a** SEM image of shear bands upon bending the Fe_78_Si_9_B_13_ MG ribbons. **b** 3D AFM topographic image of shear bands. **c**, **d** AFM topographic image (**c**) and the corresponding MFM phase image (**d**) of a single shear band. For MFM phase image, wave-like magnetic domain patterns are formed along the shear band with the light white (dark blue) regions corresponding to the magnetization pointing out of (into) the sample plane, respectively. **e** Height and the corresponding MFM phase profiles along the transverse black and red dotted lines in **c** and **d**, respectively. **f** MFM phase profiles along the lines on both sides of the shear band, as indicated by the colored dotted lines in **d**. **g**, **h** AFM topographic image (**g**) and the corresponding MFM phase image (**h**). For MFM phase image, wave-like magnetic domain patterns can extend tens of micrometers. **i** Height and the corresponding MFM phase profiles along the transverse black and red dotted lines in **g** and **h**, respectively. **j**, MFM phase profiles along the colored dotted lines in **h**. The intensity of the extending magnetic domains (blue line) is much weaker than the domains surrounding the shear band (red line). Scale bar, 5 µm in **a**, **c**, **d** and 10 µm in **g**, **h**

The commercially available Fe_78_Si_9_B_13_ MG ribbons were used in this study. We created shear bands by bending the ribbons to 180° [Refs.^29–31^] and the spacing of shear bands was controlled by bending radius. The free surface that underwent compressive stress was chosen for shear banding study. Fig. 1a shows a typical scanning electron microscope (SEM) image of shear band morphology after deformation. The shear bands are in parallel to the bending axis, and the spacing between adjacent shear bands ranges (Fig. 1a, as an example, shows the spacing between adjacent shear bands ranges from 1 to 6 µm), then we can study a single shear band or multiple shear bands. The shear steps caused by shear slipping range from 29 to 44 nm as revealed by atomic force microscope (AFM) topography (Fig. 1b). It should be noted that no cracks are observed in all AFM images.

Fig. 1c, d show the topography of a single shear band and the corresponding MFM phase image, respectively. From the height profile (see the black line in Fig. 1e), the shear step can be found to be 18-nm high for the shear band. In the MFM phase image, wave-like magnetic domain patterns are observed on both sides of the band. As shown by the phase profiles along the shear band, evident out-of-plane magnetization components, alternately pointing to opposite directions, can be found (Fig. 1d, f). For materials with isotropic magnetostriction constant *λ*, the magnetoelastic energy, *E*_ME_, can be expressed as $E_{{\mathrm{ME}}} = - \frac{3}{2}\lambda \mathop {\sum }\limits_{i = 1}^3 \sigma _i\gamma _i^2$, in which *σ*_*i*_ is the applied stress and *γ*_*i*_ is the direction cosine of the magnetization vector with respect to the stress axis^26,32^. Since the Fe_78_Si_9_B_13_ MG exhibits a positive *λ* (*λ* = 27 ppm), the magnetization direction tends to be along with the tensile stress direction or perpendicular to the compressive stress direction to minimize the magnetoelastic energy. Here, since no external magnetic field has been applied, the perpendicular magnetization component against demagnetization is thus caused by out-of-plane tensile stress though inverse magnetostriction effect. Therefore, the distribution of magnetic domains is a direct reflection of the stress field around the shear band. The non-uniform domain patterns across the shear band suggest that the stress field is discontinuous from one side of the band to another. As shown in Fig. 1d and MFM phase profile in Fig. 1e, the magnetic domains span a width of about 5 µm, suggesting the width of the SBAZ. We note that our observations on the SBAZ width are consistent with those revealed by hardness measurement^11^ and X-ray strain mapping^15^, indicating that magnetic domains are excellent tools for visualization of affected zones around shear bands.

In addition to the wave-like domain patterns in the vicinity of a shear band, extending domains in a wider region away from the band can also be observed. As shown in Fig. 1g, h, wave-like domain patterns form closely to the shear band. But at some locations, there exist bigger domains extending tens of micrometers away from the shear band, suggesting an extended stress or strain field upon shear banding (see also Supplementary Fig. 2b). The out-of-plane signal of the extending domains is much weaker than that of the wave-like ones (see the blue and red lines in Fig. 1j), implying that the SBAZ can be considered as the severely deformed zone (several micrometers in width) along with long-range strain gradient field (tens of micrometers in width). This indicates that shear banding can affect a much wider region away from the shear band.

**Fig. 2:**
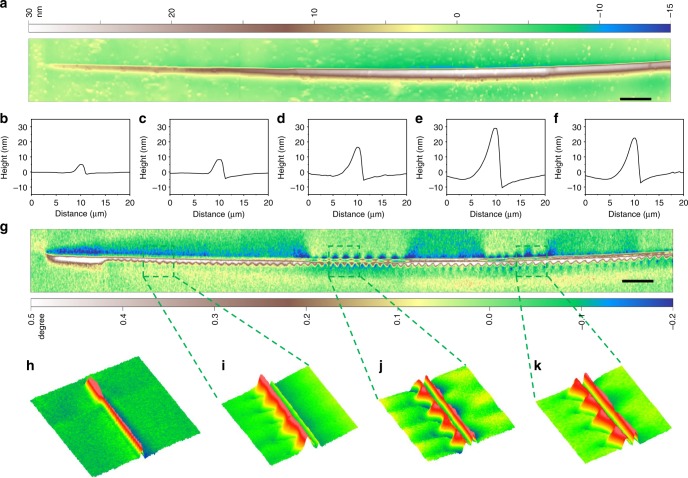
AFM and MFM micrographs of a 200-μm long shear band. **a**, **g** AFM topographic image (**a**) and the corresponding MFM phase image (**g**) covering 200 µm for a single shear band. **b**–**f** Height profiles at different locations along the shear band. **h** 70 × 70 µm^2^ 3D MFM phase image at shear band propagation front. **i**–**k** Zoom-in 3D MFM phase images in the 10 × 10 µm^2^ regions marked by the green squares in **g**. Scale bar, 10 µm in **a** and **g**

To reveal the evolution of SBAZs with shear banding, we scanned a 200-μm-long shear band till the propagation front. Fig. 2a, g show the topography and MFM phase image, respectively. As can be seen, the height of the shear step continuously decreases from tens of nanometers to zero at propagation front. This indicates that the propagation of shear bands is by a progressive manner upon bending, similar to that reported upon compression^33^. Along the shear band, with the decrease of the shear step height, the width of wave-like magnetic domain patterns gradually decreases from 3 to 1 µm, as shown by the zoom-in 3D images in Fig. 2i–k and Supplementary Fig. 1. However, at the propagation front (Fig. 2h), it appears that the intensity of out-of-plane magnetization becomes significantly stronger and the width of the magnetic domain pattern becomes much wider (see also Supplementary Fig. 1). This observation indicates that a strong stress concentration is required for a shear band to propagate, in agreement with the rejuvenation at the shear-band front, where the glass undergoes a transition to a higher-energy state under a locally high stress^1,34^.

Generally, a shear band has been considered to be initiated from cooperative rearrangements of numerous STZs^35^. The activation of STZs induces elastic displacements in the surrounding matrix leading to “Eshelby backstress”^36–40^. The cooperative local STZ events produce long-range elastic stress fields with measurable effects, resulting in an overall macroscopic strain^1,38,39^. From this point of view, it is reasonable to assume that the wave-like magnetic domain patterns, originating from magneto-elastic coupling, is the result of elastic atomic displacements due to STZ activation. In other words, STZs occur not only in the nanoscale shear-band core, but also in the SBAZ surrounding shear bands, in good agreement with the simulated distribution of STZs^5,16^. In previous studies, attention is mainly paid to the primary shear band that shows large shear offsets and carries nearly all the macroscopic plastic flow^10–13^. However, we carried out the magnetic domain imaging on regular shear bands. Our observations indicate that the micrometer-scale SBAZ is not limited to any specific shear bands but a ubiquitous feature of shear banding.

**Fig. 3:**
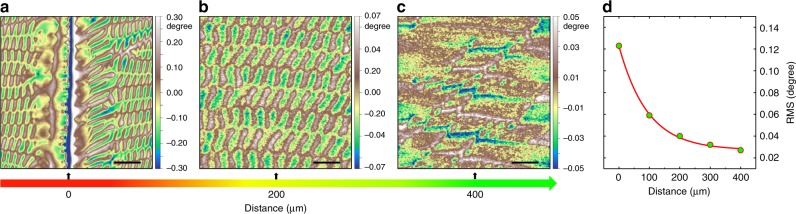
Ultra-long-range magnetic domain patterns around a single shear band. **a**, **b**, **c** Typical MFM phase images at different distances from the shear band. **d** Root mean square (RMS) values of the MFM phase shift as a function of the distance from the shear band. Scale bar, 5 µm in **a**–**c**

Surprisingly, in addition to the above wave-like magnetic domain patterns, ultra-long-range zipper-like magnetic domain patterns are also observed. Fig. 3a–c shows the evolution of magnetic domain configurations with increasing distance from a shear band. In the vicinity of the shear band, the wave-like magnetic domain patterns still exist (Fig. 3a). However, at further distance, the magnetic domains evolve to zipper-like patterns. At the distance of 200 µm, the zipper-like patterns emerge with their intensity exhibiting pronounced decreases (Fig. 3b). At even further distance up to 400 µm, the zipper-like domains evolve to irregular zigzag patterns and their intensity fades out (Fig. 3c). To quantify the evolution, we use the root mean square (RMS) values of the phase shift of the MFM phase images as a measure of the magnetic domain intensity. As shown in Fig. 3d, the RMS of the MFM phase decreases with the increasing distance from the shear band. The variation of the magnetic domains visually presents the gradual evolution of the long-range stress field that can extend hundreds of micrometers far beyond the shear band.

The distinctly different magnetic domain configurations (the wave-like domain patterns and the zipper-like domain patterns) suggest their different origins. The SBAZs reflected by wave-like magnetic domain patterns, spanning a few to tens of micrometers in width in the vicinity of shear bands, forms accompanying the formation of shear bands. They contribute to strain localization of MGs during plastic deformation, indicating the effective deformation zone of shear banding or the effective thickness of shear bands^41^. The ultra-long-range zipper-like magnetic domain patterns suggest the much wider SBAZ than expected before. We note that the zipper-like domains only appear when nearly all plastic deformation is localized into a few shear bands. Since the perpendicular magnetization component of the zipper-like magnetic domains arises from in-plane compressive stress, they reflect the existence of an ultra-long-range elastic deformation regime frozen around the shear band upon unloading. Before the formation of a shear band, a long-range elastic stress field builds up. Once a shear band forms, the elastic stress field redistributes and forms the long-range gradual stress field extending up to hundreds of micrometers away from the shear band. More shear bands are initiated upon yielding and the elastic energy in the elastic regime dissipates in shear bands, as well as their surrounding matrix, resulting in the wave-like domain patterns surrounding shear bands. Considering the fact that non-affine atomic displacements and shear transformations occurring within the elastic regime^41–45^, properties associated with microstructure in the ultra-long-range elastic deformation regime will also be influenced. The mentioned some hundreds of micrometers wide soft zone interpreted as long-range stress fields around a single shear band that carried all the plastic strain^12,13^ is in agreement with the ultra-long-range elastic regime we observed by magnetic domains. Whereas our mapping of the evolving zipper-like domain patterns provides a visualized look at the apparent long-range elastic regimes around shear bands, and unveils the gradual evolution of the elastic stress fields.

### Magnetic domains around multiple shear bands

**Fig. 4:**
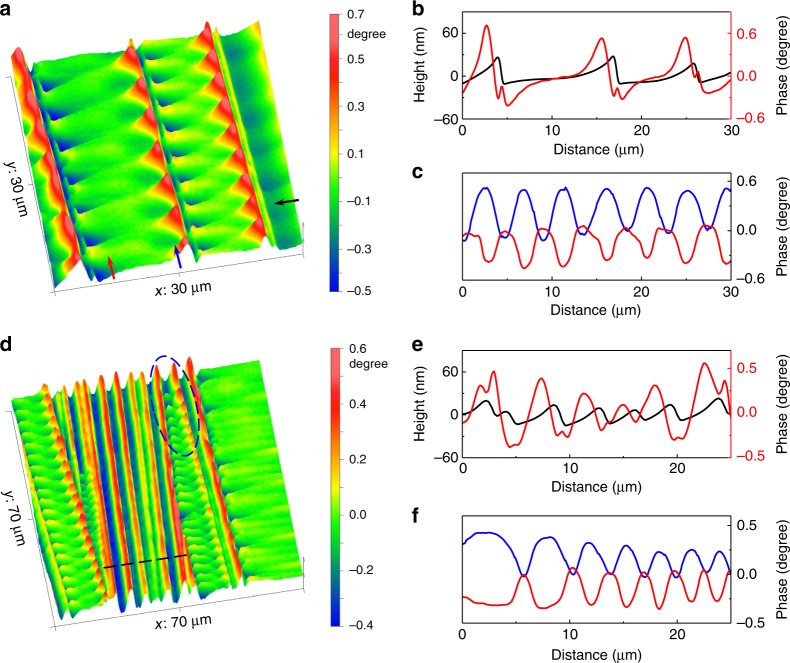
Typical MFM micrographs of multiple shear bands. **a** MFM phase image of multiple shear bands. **b** Height and the corresponding MFM phase profiles along the path indicated by the black arrow in **a**. **c** MFM phase profiles along two adjacent shear bands indicated by the colored arrows in **a**. The magnetic domains between adjacent shear bands appear in pairs, resulting in domains of into-plane magnetization near one shear band correspond to domains out-of-plane magnetization near the other shear band. **d** MFM phase image of multiple shear bands with intersections. **e** Height and the corresponding MFM phase profiles along the line in **d**. **f** MFM phase profiles along two intersecting shear bands in the region marked by the blue ellipse in **d**

Shear band multiplication and interaction play vital roles in enhancing deformability of MGs. However, due to technical limitations, little work has been done on the SBAZs around multiple shear bands. Fig. 4a shows an MFM phase image at region where multiple parallel shear bands form. Compared with the case of a single shear band (Figs 1d, 2g), the wave-like magnetic domain patterns appear to be larger in size (Fig. 4a), suggesting a wider SBAZ in the case of multiple shear bands. MFM phase profile in Fig. 4b shows that the out-of-plane magnetization intensity gradually weakens. This indicates that there is a gradient in the strain field. Furthermore, the wave-like domains between adjacent shear bands occur in pairs, i.e., domains of into-plane magnetization near one shear band correspond to domains out-of-plane magnetization near the other shear band, as shown in the MFM phase image and phase profiles (Fig. 4a, c). The occurrence of paired magnetic domains reflects that the stress fields in their respective SBAZs are superimposed in the region between them (see also Supplementary Fig. 2). These observations demonstrate that the interaction of shear bands is in fact the superposition of stress fields created in the SBAZs.

Shear band intersection is a common phenomenon, particularly, in MGs with large plasticity. Fig. 4d presents the appearance of magnetic domain patterns at region where shear band intersection occurs. As can be seen in Fig. 4d, at intersections, the propagation of shear bands is suppressed by those shear bands propagating to another direction. As indicated by the blue ellipse and the phase profiles (Fig. 4d, f), with the decreasing spacing between two intersecting shear bands, the magnetic domains evolve regularly with the size of paired domain increase (see also Supplementary Fig. 2f). This is attributed to the increased stress field superposition from the SBAZs at reduced shear band spacing. In addition, it can also be seen that when the shear band spacing is smaller than the width of SBAZs, wave-like domain patterns change to stripe-like domains, as marked by the black line in Fig. 4d. In this case, one shear band is in the effective deformation zone of the adjacent shear band, in line with the prediction that the operation of adjacent shear bands is mutually affected^1^. From the observations on multiple shear bands, it can be deduced that each shear band is surrounded by a micrometer-scale effective deformation zone or SBAZ, and the nature of shear band interaction is the superimposition of stress fields in the SBAZs.

**Fig. 5:**
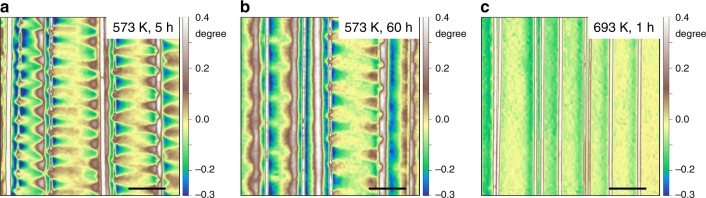
Evolution of magnetic domains after annealing. **a** MFM phase image after annealing at 573 K for 5 h. **b** MFM phase image after annealing at 573 K for 60 h. **c** MFM phase image after annealing at 693 K for 1 h. The white lines, caused by the jump of the MFM tip, stand for the shear band positions. Scale bar, 10 µm in **a**–**c**

We note that the magnetic domains around shear bands have no obvious change after the sample being kept for more than 5 months at room temperature, verifying the good magnetic stability in the SBAZs. To further confirm the stability of magnetic domains, the deformed samples were annealed at different temperatures well below the onset crystallization temperature (see Supplementary Fig. 3). As illustrated in Fig. 5a, after annealing at 573 K for 5 h, the magnetization contrast of paired wave-like magnetic domains become relatively weaker, arising from annealing induced stress relaxation. After 60 h annealing, the magnetic domains still persist (Fig. 5b). This implies that synergy between atomic structural changes and elastic displacements in the SBAZs contributes to the formed domain configurations around shear bands, e.g., STZ activation induced structure changes and elastic backstress^16,38–40^. After annealing at 693 K (about 18 K above the Curie temperature *T*_c_) for 1 h, the magnetic domains are completely erased (Fig. 5c), indicating the lowest-energy configuration of magnetic domains have changed. This arises from structural relaxation induced atomic rearrangement upon annealing approaches the glass transition temperature^46,47^, consistent with the disappearance of the soft region around the shear band, which is interpreted as the annihilation of free volume by annealing^10^.

## Discussion

Taking magneto-elastic coupling induced magnetic domains as visualization tools, we have demonstrated the SBAZs from the cores to far away from shear bands. Compared with the recent investigations by nanoindentation^10–13^ or nanobeam X-ray diffraction^15,16^, our mapping of magnetic domains is of much higher spatial resolution and more sensitive to the displacements of atoms. This enables us to precisely identify SBAZs with different length scales. We unveiled two types of affected zones around shear bands. One is the effective deformation zone of shear banding, reflected by wave-like magnetic domain patterns in the vicinity of a shear band. The other is the ultra-long-range elastic regime, reflected by zipper-like domain patterns extending up to hundreds of micrometers away from the shear band.

**Fig. 6:**
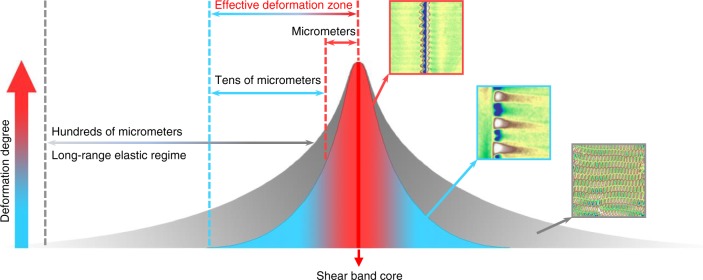
Schematic of the shear-band affected zone. The gray region represents the hundreds of micrometers long-range elastic regime. The colored region stands for the effective deformation zone of shear banding: the bright red line stands for the core of a shear band with nanoscale; the red region represents the severely deformed zone with several micrometers in wide; the light blue region represents the extended strain gradient field with tens of micrometers long. The three 30 × 30 µm^2^ MFM phase images stand for the typical magnetic domain patterns (wave-like domain pattern, extending domain pattern, zipper-like domain pattern) in the corresponding regions

From the magnetic domain configurations and their evolution, the SBAZ appears to have a multilayer-like structure, as schematically illustrated in Fig. 6. During plastic deformation, the nanoscale core of the shear band (see the red line in Fig. 6, as observed by TEM^29,30^) carries a significant portion of plastic strain. Nearby the shear-band core, there is a region that undergoes severe deformation (the red region in Fig. 6), corresponding to the wave-like magnetic domains. This region spans a few micrometers from the core. Beyond the severely deformed zone, a strain gradient field forms and extends tens of micrometers upon shear banding (the blue region in Fig. 6), corresponding to the extending wave-like magnetic domains. The whole colored region presents the effective deformation zone of shear banding, which unravel the localized nature of shear banding is far from being limited to the nanoscale core of a shear band. The paired wave-like magnetic domain patterns between adjacent shear bands give an ocular correlation of multiple shear bands. This correlation arises from the superposition of stress fields extending from their respective shear bands. In brief, multiple shear bands interact through the overlap of their respective effective deformation zones, avoiding plastic flow only in a single shear band and causing catastrophic avalanche. The hundreds of micrometers long gray region in Fig. 6 stands for the ultra-long-range elastic regime, which is particularly evident surrounding shear bands that carry large plastic flow. Stress field in the elastic regime evolves gradually and extends hundreds of micrometers away from the shear band. This picture of the SBAZ based on our magnetic domain observations explains the various affected zone widths ranging from submicrometer to hundreds of micrometers reported in previous investigations^10–17^. It should be mentioned that the multilayer-like picture is only for easy description of the SBAZ. There should be no sharp interfaces separating the zones, and the variation from one layer to another should be continuous.

Based on our observations on the SBAZs, many previously reported phenomena associated with shear banding can be well explained. For example, it is known that MGs can store a lot of elastic energy due to their low Young’s modulus and large elastic limit^1^. The existence of an ultra-long-range elastic regime indicates that the strain energy can be stored in a wide region. Rejuvenation achieved by plastic deformation or the stored energy of deformed MGs cannot be attributed only to the nanoscale shear band^4,43^, because atomic structural changes induced by shear banding is in a much wider region than the core, and it is the whole micrometer-scale effective deformation zone of shear banding, which takes a much larger volume fraction, is responsible for the change of the energy state of deformed glasses. This explains why there is a significant discrepancy between the experimentally obtained stored energy and the estimated value with shear band thickness being 20 nm^4,43^. A typical feature of the fracture surfaces of MGs is the existence of a liquid-like layer with a thickness of a few micrometers. According to our findings, such a liquid-like layer is the consequence of the micrometer-scale effective deformation zone including the shear-band core, rather than the nanoscale core itself^1,3,8^, which is critical for understanding the fracture mechanism of MGs. Moreover, the SBAS illustrated in Fig. 6 also indicates that the enhanced relaxation dynamics such as the diffusion enhancement behavior^5^ and the accelerated aging processes^14^ in deformed glasses should arise from a much wider region than the shear-band core.

In summary, by using magnetic domains as a probe, we demonstrate the structure of shear banding induced SBAZs with high sensitivity and spatial resolution. We found that the SBAZ is composed of the nanoscale shear band, the micrometer-scale severely deformed zone in the vicinity of the shear band, and the tens of micrometers extended strain gradient field. With the decrease of shear band spacing, it is the SBAZ of each band that results in shear band interaction. There also exists an ultra-long-range elastic regime with gradual stress field extending up to hundreds of micrometers from the shear band. Our method and findings provide a visualizable insight into SBAZs and enable a comprehensive understanding of strain localization in MGs. The revealed SBAZs are important for understanding the microscopic mechanisms of plastic deformation in MGs and thus for the design of tough MGs.

## Methods

### Sample preparation

The Fe_78_Si_9_B_13_ MG ribbons with 25 µm thick were prepared by melts pinning in argon atmosphere. Before further experiments, the ribbons were ultrasonically cleaned in acetone and ethanol, and blow-dried with a nitrogen gun. Bending was performed using two parallel plates fixed at the two sides of a vernier caliper. Upon decreasing the spacing between the plates, the ribbons were bent to 180°. When the bend radius was ~1 mm, plastic deformation took place. Then the ribbons were released from the plates.

### MFM measurements

Atomic and magnetic force microscopy measurements were carried out with an Asylum Research MFP-3D AFM (Asylum Research). Commercially available magnetic tips (Nanosensors, PPP-MFMR, resolution <50 nm) were used to record the local magnetic domain configurations. MFM phase images were acquired simultaneously with AFM images using the standard two-pass technique: the first pass was performed to record the topography in intermittent contact mode; the second pass was performed to record the magnetic phase shift by keeping the tip at a selected lift height with respect to the recorded topography. In our study, the magnetic tip was kept at a lift height of 100 nm to avoid topographic artifacts.

## Electronic supplementary material

Supplementary Information

## Data Availability

The data that support the findings of this study are available from the corresponding author upon reasonable request.
