# Supplementary material for: Shear-band affected zone revealed by magnetic domains in a ferromagnetic metallic glass
Source: Nat Commun. 2018 Oct 24;9:4414. doi: 10.1038/s41467-018-06919-2 (PMC6200802; doi:10.1038/s41467-018-06919-2)
Supplement: Supplementary file 1 — Supplementary Information [file 41467_2018_6919_MOESM1_ESM.pdf]

Supplementary Information for

Shear-band affected zone revealed by magnetic domains in a ferromagnetic metallic glass

Shen *et al*.

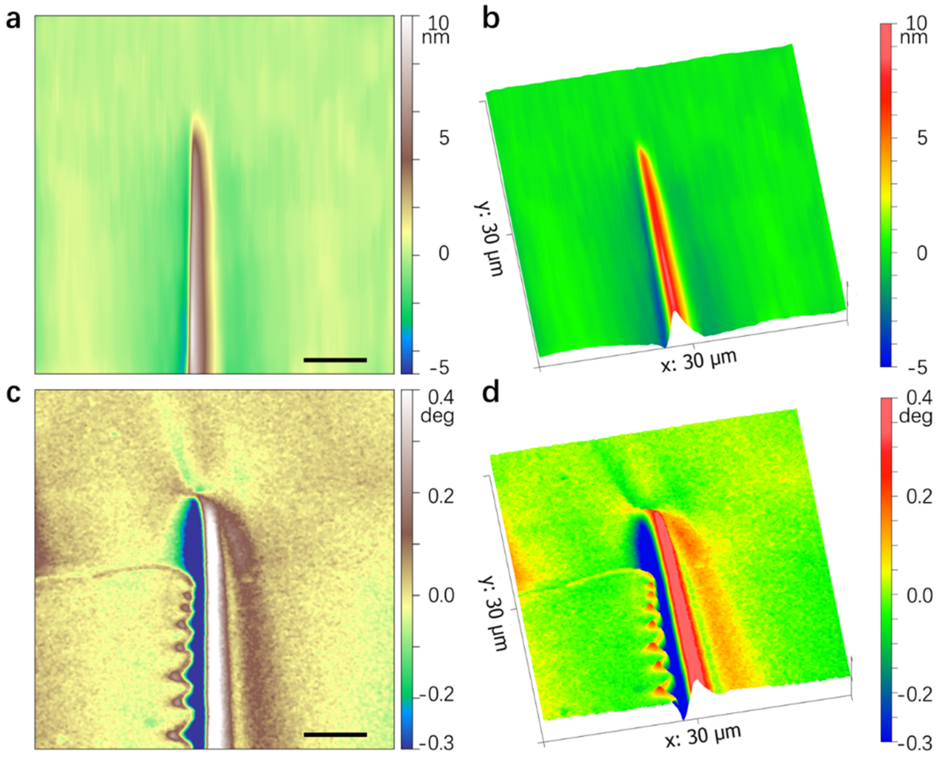

Supplementary Figure 1. AFM and MFM micrographs of shear band propagation front. **a**, **b**, AFM topographic image (a) and the corresponding 3D topographic image (b) of the shear band propagation front. Height of the shear step continuously decreases to zero at the propagation front. **c**, **d**, MFM phase image (c) and the corresponding 3D phase image (d) of the shear band propagation front. It is clearly seen that the size of the wave-like domain patterns becomes narrower with the shear step height decreasing, but around the propagation front, the domain size suddenly become larger, indicating a stress concentration around the propagation front of the shear band. Scale bar, 5 µm in **a**, **c**.

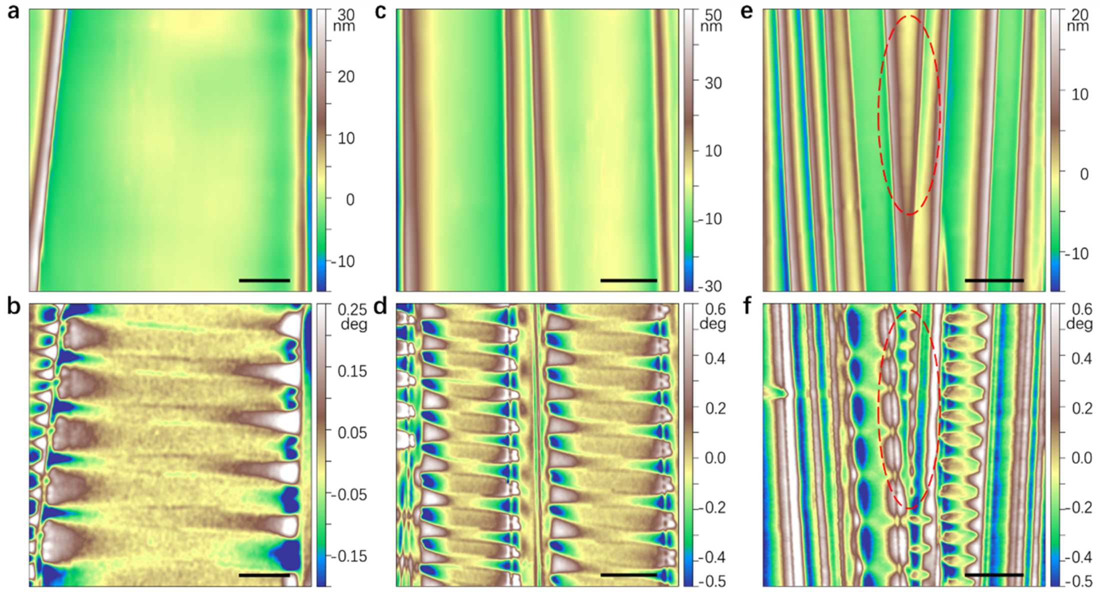

Supplementary Figure 2. AFM and MFM micrographs of multiple shear bands. a, **b**, AFM topographic image (a) and the corresponding MFM phase image (b). The spacing between the adjacent shear bands in Fig. a is more than 50μm, but the paired magnetic domain patterns can still exist (Fig. b). It is clearly seen that the wave-like domain patterns can extend tens of micrometers and the out-of-plane signal of the extending domains gradually weakens, similar to the situation in Fig. 1h. Figure b also present that the paired domains between the adjacent shear bands result from the superimposition of the extending domains from their respective wave-like domains, indicating that the stress fields from their respective shear bands are superimposed. AFM topographic image (**c**) and the corresponding MFM phase image (**d**) present the typical paired magnetic domain patterns between adjacent shear bands. **e**, **f**, AFM topographic image (e) and the corresponding MFM phase image (f) of multiple shear bands with intersections. As marked by the red ellipse, with the decreasing spacing between two intersecting shear bands, the magnetic domain patterns evolve regularly, resulting from the increased stress field superposition from their respective shear bands with the decreasing shear band spacing. Scale bar, 10 μm in **a-f**.

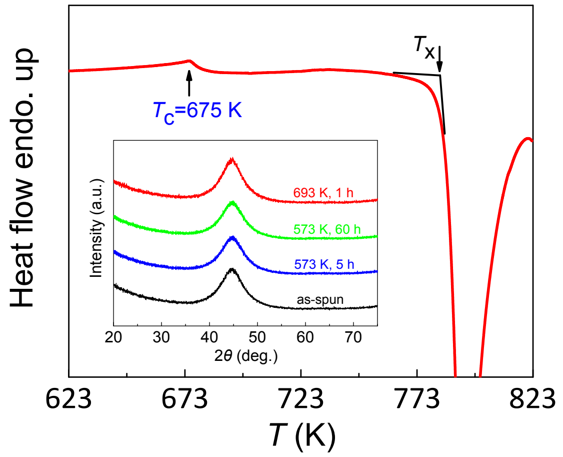

Supplementary Figure 3. DSC and XRD results. Representative DSC trace at a heating rate of 20 K min^{-1} for the $Fe_{78}Si_9B_{13}$ MG ribbon. The arrows designate the Curie temperature T_c and the onset crystallization temperature T_x. Inset: XRD patterns of the as-spun and annealed samples.
